# Injuries to the Head Illustrating Functions of the Cortex

**Published:** 1923-07

**Authors:** R. G. Gordon

**Affiliations:** Neurologist, Ministry of Pensions, Bath; Physician, Royal Mineral Water Hospital, Bath


### INJURIES TO THE HEAD ILLUSTRATING FUNCTIONS OF THE CORTEX.

BY

R. G. Gordon, M.D., M.R.C.P. (Edin.),

*Neurologist, Ministry of Pensions, Bath ; Physician, Royal Mineral Water Hospital, Bath.*

The whole subject of cortical function is beyond the scope of a single paper, and all I shall attempt to do is to illustrate some of the recent work on the subject from cases which have recently come under my observation.

To begin with, it must be remembered that the nervous system should be thought of as a hierarchy of arrangements of neurones all modelled on the simple reflex arc. This is true whether we consider this arrangement from the point of view of structure, that is to say, receptors, central neurones, muscles and glands ; or of physiological function, which involves reception of stimulus, activation of neurones, muscular or glandular activity ; or of the psychical accompaniments of these, namely sensation, perception, feeling and desire. At any level in this hierarchy we find that a pattern of neurones and their connections such as that described above controls analogous patterns below it in the scale, and is itself controlled by analogous patterns above it.

Sherrington[10] showed how the nervous system may be divided into two very distinct parts. Firstly, that subserving the response to those stimuli which required intimate contact with the body to register their impression, such as touch, pain, heat and cold, etc. This is the spinal part, comprising the spinal cord and brain stem. Secondly, that subserving the response to stimuli acting at a distance, *i.e.* light, sound, and (with certain reservations) smell. This part is the brain proper. Of this latter part phylogenetic and ontogenetic evidence shows us that the cerebral cortex constitutes the newest, most complicated and highest series of patterns in the hierarchy.

Recently Bianchi[1] has shown that of the cortical areas the frontal lobes contain the patterns which integrate and control the others. Another aspect is, however, of importance. If the various areas in the cortex are examined six layers of cells may be discovered[7] making up the superficial layer of grey matter, and there is considerable evidence to show that, at any rate so far as local function is concerned, the function of the superficial layers is to control that of the deeper ones.

Rivers,[8] Head,[3] Sherrington[11] and others have very clearly demonstrated how the function of a lower pattern is modified by that of higher patterns. It may be altogether suppressed, so that no trace of its activity is disclosed until it is released by interference with the activity of all higher patterns. An example of this is afforded by the discovery by Head and Riddoch [5] of the mass reflex in cases which recovered from complete division of the spinal cord. This reflex consisted in the response to a stimulus of the foot or any part of the leg by flexion, sweating, and voiding of the bladder and rectum. Such a reflex may be very useful to certain molluscs such as the squib, but is of no use to man. Or the function of the lower pattern may be fused with that of the higher, so that it forms an essential part of that function. An example of this may be seen in the maintenance of normal muscular tone, which is profoundly disturbed if the function of the lower pattern is interrupted by lesions of the lenticular nucleus, though the higher cortical pattern is intact. This state of affairs is seen in paralysis agitans and allied diseases.

A factor of great importance to remember when considering this hierarchy of patterns is that as we rise in the scale not only do the patterns increase in complexity but also in delicacy of adjustment, so that (if one may be permitted a hyperbole) a crowbar is needed to disturb function at the bottom of the scale but an angry word is sufficient at the top.

Another function of the cortex which must be realised is the so-called retention of memory, which involves the power of recalling images. Suppose a group of stimuli affects the brain ; for example, the light waves from a tree impinge upon my retina and are conveyed to my occipital cortex, my experience is that I see the tree, but the activation of these various neurones which takes place does not leave them the same as before. What is called an engraphic effect [9] has taken place, and that particular arrangement of neurones tends to react in the same way in future in response to similar stimuli. In this way images of the tree may be aroused, irrespective of the actual presence of the tree. It is the function of the cortex to correlate any given sensation with revived images of similar sensations, and so to give meaning to the whole and enable us to exercise what is called perception. This involves discrimination and integration of the images and sensations. It is the function of the cortex, then, to receive impressions from those sense organs reacting to stimuli at a distance, to correlate all sensory messages with images of both similar and dissimilar sensory impressions, and to discriminate between them, thereby permitting reference in time and space; it exercises control over the functions of lower levels in the hierarchy; and, finally, here are integrated all impressions in such a way as to subserve the intellectual life of the individual and to discriminate and control efferent impulses so as to allow of the most delicate manipulative activities.

The object of this paper is to try to illustrate these functions by reference to the effect of injury of the superficial cortex in the occipital lobe, the sensory area, the motor area and the frontal lobes.

**The Occipital Lobe.**—The first case was that of a man who suffered a severe blow on the back of the head from a fall from a lorry travelling at a high speed. The injury, which was probably a subdural hemorrhage, had involved the whole of the area of the visual cortex on both sides, with the result that he was completely and hopelessly blind; but as the mid-brain centres for muscular movements were uninjured there was no ophthalmoplegia. In other words, the more distal part of the optic tract was intact. This illustrates the function of the cortex as a receiving station for sensory impressions acting at a distance.

The second case was that of a man who was injured by a gun-shot wound on one side only, resulting in the patient suffering from a homonymous hemianopsia. This illustrates the principle that we shall find more evident later, that the cortex has to do with physiological function rather than anatomical structure. Thus the left occipital cortex has to do, not with the right eye, but with the perception of stimuli coming from the right side ; just as we shall find that the motor cortex has to do with movements and not with anatomical muscular groups.

The third case was one of a large gun-shot wound on the lateral aspect of the occipital lobe, which must have caused injury to a considerable area of the cortex, though it did not involve the actual visual area. This man had been a ship's purser, and had travelled widely, knowing several languages. He still retained considerable intelligence, and so his condition was easy to investigate. Before the injury to his brain his visual imagery had been extremely accurate and vivid. Now it was with the greatest difficulty that he could give any description of what to him had been a perfectly familiar sight, namely the rock and harbour of Gibraltar. He had only a vague idea of the shape of the rock, and could get no picture of the sweep of the bay or where Algeciras lay. Even such a familiar object as Clifton Suspension Bridge was described but vaguely, with no detail whatever. His languages were very much impaired as to vocabulary, though his accent, which depended on auditory imagery, was fair. This illustrates the function of the cortex in revival of images.

A fourth case was that of an epileptic whose fits dated from the age of six, when he sustained a severe injury of the right temple. At times the ordinary attacks of grand mal and petit mal were replaced by what are called visual equivalents. In these attacks there appeared before his eyes lights or sometimes figures which started in the centre of the field of vision and passed over to the right margin of the field. While these were going on he had a right homonymous hemianopsia, which passed off with the end of the attack. Here there was suppression of the proper highest function of the visual cortex at the left occipital pole with substitution or liberation of lower and less co-ordinated function. This corresponds exactly with what happens in lesions of the motor cortex which cause fits. The cause of these attacks is doubtful, but I venture on this explanation. At the time of the blow there was a disturbance at the opposite occipital pole by contre-coup, which injured the superficial cells of the left occipital lobe, so that under certain conditions their function was entirely abrogated, and the activity of deeper and lower grade cells was liberated.

In this connection a result of less severe occipital lesions cannot escape notice. That is the frequency with which typical migrainous attacks are initiated by such an injury. These are associated with a visual aura followed by headache, and the analogy with the visual epileptic equivalents, described above, is at least permissible. I would suggest that these attacks of migraine depend, at least partially, on an interference with the highest grade superficial cells whose function it is to control the activity of the lower; further, that a liability to migraine depends on an instability of these highest grade occipital cells, just as an epileptic fit depends on an instability of the highest grade cells of the motor cortex. This, of course, does not explain the onset of any individual fit or attack of migraine, but only the tendency of the patient to suffer from them.

As to the discriminative function of the occipital cortex, this is probably concerned with colour vision. It is a matter of common knowledge that savages are often unable to discriminate between different shades of the same colour, and the same applies to many low-grade intellects in civilised communities. Presumably colour blindness may have something to do with congenital interference with this discriminative function where the receptive end organs are intact. I am not aware that any exact observations have been made on colour discrimination in connection with injuries to the occipital cortex. Holmes[6] described a doubtful case of achromatopsia with retention of the field for white, but drew no conclusions from it. However, if we remember that the higher discriminative neurones have their projection fibres proceeding from the periphery, and that the highest grade cell and fibre are always affected first in any disease process, it is a significant fact that in pituitary tumours a hemiachromatopsia often precedes hemianopsia. More work is needed on this point, which seems to have escaped the attention of neurologists and ophthalmologists.

**The Sensory Area.**—In the fifth case, which is illustrative of a large group of head injuries, there was a wound in the parietal bone over the post-central gyrus where the sensory area of the cortex is situated. We find that quite a small area of the body is affected, namely the right hand; that the patient complains of no pain, but states that he has a "paralysis" of his right hand. When we examine him, however, we find there is no paralysis and that the muscles work all right, with certain reservations. If both hands are stretched out in front of him there is a slight drooping of the right; he has a mild degree of hypotonia which is really due to false co-ordination. If he is asked to perform certain movements requiring manipulative dexterity, such as opposing the thumb to the other fingers in succession, picking up a pin or doing up a button, he is hopelessly at fault. If we examine his sensation we find he can appreciate pain, extremes of heat and cold and touch, but he cannot localise where the stimulus was administered with any degree of accuracy, nor can he discriminate the exact nature of the stimulus, and above all he has no idea of the position of his hand or fingers. He shows partial astereognosis, and fails to distinguish between the different textures of materials, such as silk and cotton. The result of all this is that, in spite of appearances, he has a very inefficient hand, and except for steadying things he might just as well be without it.

To explain why such a state of affairs is met with we must consider for a moment the physiology of the sensory paths. All the various forms of common sensation are subserved by special end organs in the skin and deeper parts, but all similar sensory paths are quickly grouped together in the spinal cord. Those for pain, heat and cold and certain touch fibres pass to the opposite side of the cord and travel up the lateral columns to the corresponding optic thalamus. The fibres concerned with posture, discrimination between two compass points and the rest of the tactile fibres proceed up the posterior columns of the same side to the nuclei of the fillet in the medulla. Thence fibres cross to the opposite side and travel to the optic thalamus. Here, then, all sensory fibres from the opposite side of the body meet once more at their second cell station within the central nervous system. Thence the sensory impulses are redistributed. All or almost all sensations have two aspects, a discriminative and an affective aspect. By the first we mean the reception of the information which would answer the questions, Where in the body was the stimulus applied which gave rise to this sensation ? What sort of a sensation was it ? and What sort of a thing caused it ? By the second or affective aspect we mean the reception of information telling us what it feels like, was it pleasant or unpleasant ? Head [4] has shown that this latter affective aspect is dealt with by the lateral nucleus of the optic thalamus, while the former discriminative aspect is dealt with by the cortex. It is obvious that such a sensation as posture has little or no affective aspect ; for instance, it is not specially pleasant or unpleasant to have one's fingers bent or straight, and correspondingly this sensation is not represented in the thalamus, nor affected in lesions of this. On the other hand, such a sensation as pain is almost entirely affective, and in consequence it is not represented on the cortex to any extent, and in lesions of this part pain is not interfered with. The lateral nucleus of the thalamus may be regarded as the highest station in connection with pain, and this, of course, is not easily influenced by any means physical or psychical. That is why once a real habit of pain has been established it is so extremely difficult to get rid of, though in less severe cases functional pain may be readily removed.

The sensory cortex subserves five functions : (1) the recognition of posture and passive movement, (2) the recognition of certain tactile elements ; (3) the appreciation of two points applied simultaneously to two different points on the surface of the skin, and also the recognition of the size and shape of objects which are in contact with the skin (spatial discrimination) ; (4) the localisation of the situation of a stimulated spot on the skin and the recognition of two consecutive stimuli (discrimination of time) ; (5) the recognition and discrimination of a scale of sensations with heat at one end and cold at the other. In addition, it is the function of the cortex to relate one sensation with another. We see that these functions correspond to the deficiencies exhibited by the patient whose case is described above. Graham Brown and Stewart[2] experimented on the retraining of tactile localisation on such a "sensory" hand, and met with considerable success. Personally, I have not had the opportunity of carrying out retraining experiments with such exactitude as they did, but in my experience something can be done by re-education in restoring the use of the hand and a little in restoring the sense of position.

**The Motor Area.**—It is not necessary to say much about the motor cortex, which is situated in the precentral gyrus, because injuries in this area are productive of the most obvious effects, which are familiar to everyone. It should be noticed that even in cortical injuries the motor symptoms are often complicated by sensory deficiences due to injury of the adjacent sensory cortex; and in the vascular lesions so common in civil life, in which it is the subcortical fibres of the internal capsule that are affected, the sensory fibres are almost always damaged at the same time. So far as the motor symptoms are concerned we notice two main features, an alteration in tone and a special type of paralysis. The first is an increase in the normal tonic contraction of the muscles. This phenomenon has been definitely shown by animal experiment to be due to liberation of lower motor systems which are more concerned with posture than locomotion, hence the rigidity of the lower limbs in extension. The rigidity of the upper limbs in flexion is peculiar to man, and is perhaps suggestive that in his less remote ancestry this lower motor system was already adapted to hanging on to the branches of trees, at least with the arms. So in this alteration of tone we have an excellent example of the controlling and integrating functions of the cortex, for when the latter is intact this postural function is relegated to its proper place, and in no way interferes with the more delicate and important function of locomotion. Secondly, as regards the paralysis, we find on examination that there is no complete loss of motion, though certain movements are impossible, nor is there a complete paralysis of one group of muscles as is the case in lesions at lower levels. This shows us, firstly, that the motor function when at the cortical levels is concerned with movements and not with muscles, or in other words that an integration of individual muscular actions with a proper correlation between agonists and antagonists has taken place. Secondly, if we examine the movements which are possible we discover that the limb moves as a whole, and that the more distal the joint the less independent movement is possible. This illustrates the function of the cortex in allowing discriminated movements, especially of a high grade manipulative type, so that we may presume that the accomplished pianist is one who is endowed with a particularly finely-developed motor cortex in the hand area, but he also requires an adequate associative connection with his frontal lobes, and through them with his temporal lobes, if he is to correlate his finger movements with his auditory images of notes, for they are the final and highest integrative station.

A few words may be said here as to fits. Seizures due to trauma must be divided into two classes. Firstly, there is traumatic epilepsy, in which the fits resemble those of ordinary epilepsy with no definitely localising symptoms. These may develop up to two years after the injury, and tend to persist, though there may be remissions. They occur after injury in any part of the head, but most commonly after parietal injury. Thus Aldren Turner[12] describes a series of 38 cases in which 10 had frontal, 16 parietal, 9 occipital and 3 temporal lesions. As Hughlings Jackson and subsequent workers have shown, fits are a release phenomenon due to failure in the controlling function of higher cortical levels, and for this reason surgical intervention which is in any way concerned to interfere with the cortex itself is doomed to failure in these cases. The removal of a cortical scar which has already destroyed cortical tissue and allowed fits to develop will only destroy more cortical tissue and so make the fits worse, and such procedures, though all too common in earlier years, have now been given up. In traumatic epilepsy surgery has little or no place, and even such operations as filling up gaps in the skull are apt to increase the number of fits or even produce them as a new symptom.

The second class is the focal or Jacksonian fit, which always begins in some localised part and may or may not progress to complete unconsciousness. Such are due to so-called "irritation" of the motor cortex by a serous or blood cyst or spicule of bone, and come on soon after the injury. If such fits cease, as they may do, spontaneously they do not tend to recur, and they may be cured by surgical intervention to remove the source of "irritation." If, however, we retain this word irritation we must understand what we mean. The spicule of bone does not irritate the motor cortex as an electric current irritates and sends an impulse through a nerve muscle preparation, but it may produce a localised œdema whereby several cortical cells are thrown out of action, and their controlling action being removed, fits are able to occur. Removal of the local pressure allows the cortical cells to resume their function, and so re-establish control.

**The Frontal Lobes.**—This area of the brain is often described as silent; that is to say, injuries to these lobes do not produce any obvious effects. If by obvious effects are meant paralyses and sensory changes, as a rule there are none of these, but more careful investigation does elicit changes which have taken place as the result of severe frontal wounds.

I may here quote a case of a severe wound over the frontal lobes as illustrative of many. The wound was mesial in position and involved considerable destruction of brain tissue. We find the man suffering from no paralysis or sensory defects, but in certain respects he is considerably incapacitated. For example, his power of recall is very deficient, and he has great difficulty in remembering when things happened, so that in taking his history he is continually antedating and postdating his events. In other words, his power of reference in time is seriously affected. Still more, his power of correlation of ideas is at fault. He cannot grasp a story as a whole. Even in conversation he gets confused unless the statements made to him are simple and direct, yet before his wound he was an intelligent man with a good record. At the same time he has difficulty in controlling his emotions, he laughs or cries easily, loses his temper readily. If not specially aroused he is dull and apathetic, does not notice things about him, and will not readily take initiative over anything.

Such symptoms are not uncommon in all injuries to the head, but they are less definite and less persistent after wounds of other areas.

Let us turn now to the description of monkeys whose frontal lobes have been removed in the experiments carried out by Professor Bianchi,[1] who has done so much to elucidate the function of these higher parts of the brain. He finds that they show defects of perception, and of discrimination between objects with superficial resemblance; thus they cannot distinguish between threat and reality, and so become to a large extent incapable of play. They also display deficient observation, objects which would be investigated by the normal monkey being overlooked. Memory is defective, so that past experience is not utilised, and they do not learn by experience. Associative power is greatly reduced, so that there are no new adaptations. Judgment is poor and immediate. There is a lack of initiative and one movement does not lead to another; thus a monkey will seize a door handle (stimulus of bright object), but will not proceed to turn it. The primitive emotions remain, the desire for satisfaction of hunger, thirst and other organic needs, and especially that irrational, illogical fear which seems to have no definite relation to stimuli; but higher sentiments, such as friendship, gratitude, jealousy, maternal protection, dominion, authority, self-esteem, ridicule and, above all, that of sociality, disappear. Conduct is incoherent and stereotyped actions and tics are often present.

We see in the symptoms complained of by the man described above many of the characteristics of the monkeys, and they are symptoms which, though not obvious to the casual glance, are a real hindrance to anyone in the work of the world, and require to be carefully considered if an estimate is being made of such a patient's capacity to undertake employment. As was said at the beginning, the stability at these high levels is very delicately balanced, and it does not need a gun-shot wound to disturb the functions of the frontal lobes. It is a well-established rule in neurology that the most recently acquired functions and latest developed structures suffer most severely from injury or disease and probably in most cases of concussion it is the frontal lobes which suffer the chief damage and which take longest to regain their functional capacity.

The description of Bianchi's monkeys would do very well for a neurotic patient, and there can be little doubt that many of the symptoms of the neuroses are due to a functional dissociation of the frontal neurone control. The possession of well-developed frontal lobes undoubtedly allows of man's greater adaptability to the environment and elasticity of response to stimuli as compared to the animal, and we may recognise in certain savages and many defects obvious characteristics which are unquestionably due to defective development of these. In the neurotic there is no lack of development of the higher levels, but from dissociation, presumably at synaptic junctions, control is abrogated and certain functions of lower levels are allowed to express themselves unchecked, while other higher functions are split into their component parts, so that they no longer subserve a unified pattern of behaviour. However, these speculations as to the physiological explanations of the neuroses hardly have a place in this paper, whose purpose is to illustrate by the cases of injury which have been quoted the five great functions of the cortex, namely reception of stimuli acting at a distance, control, integration, discrimination and reference in time and space.

### REFERENCES.

[1] Bianchi, L., *The Mechanism of the Brain and the Function of the Frontal Lobes*, translated by Macdonald ; Edinburgh, 1922.

[2] Graham Brown, T., and Stewart, R. M., " Disturbances of Sensations in Cerebral Lesions," *Brain*, 1916, xxxix. 348.

[3] Head, H., *Studies in Neurology*, vol. ii. ; Oxford, 1920.

[4] Head, H., *Ibid.*, p. 600.

[5] Head, H., and Riddoch, G., *Ibid.*, p. 495.

[6] Holmes, G., and Lister, W. T., " Disturbances of Vision from Cerebra Lesions," *Brain*, 1916, xxxix. 34.

[7] Hoog, E. G. Van't, " On Deep Localisation in the Cerebral Cortex," *J. Nerv. and Ment. Dis.*, 1920, li. 313.

[8] Rivers, W. H. R., *Instinct and the Unconscious* ; Cambridge, 1920.

[9] Semon, R., *The Mneme*, translated by Simon ; London, 1921.

[10] Sherrington, C. S., *The Integrative Action of the Nervous System* ; London, 1906.

[11] Sherrington, C. S., *Ibid.*

[12] Turner, W. Aldren, " Epilepsy and Gunshot Wounds of the Head," *J. Neurol. and Psychopath.*, 1923, iii. 309.

